# Big Data Driven Detection of Trees in Suburban Scenes Using Visual Spectrum Eye Level Photography

**DOI:** 10.3390/s20113051

**Published:** 2020-05-28

**Authors:** Andrew Thirlwell, Ognjen Arandjelović

**Affiliations:** School of Computer Science, University of St Andrews, St Andrews KY16 9AJ, UK; at224@st-andrews.ac.uk

**Keywords:** computer vision, local features, machine learning, street view, tree stumps

## Abstract

The aim of the work described in this paper is to detect trees in eye level view images. Unlike previous work that universally considers highly constrained environments, such as natural parks and wooded areas, or simple scenes with little clutter and clear tree separation, our focus is on much more challenging suburban scenes, which are rich in clutter and highly variable in type and appearance (houses, falls, shrubs, cars, bicycles, pedestrians, hydrants, lamp posts, etc.). Thus, we motivate and introduce three different approaches: (i) a conventional computer vision based approach, employing manually engineered steps and making use of explicit human knowledge of the application domain, (ii) a more machine learning oriented approach, which learns from densely extracted local features in the form of scale invariant features (SIFT), and (iii) a machine learning based approach, which employs both colour and appearance models as a means of making the most of available discriminative information. We also make a significant contribution in regards to the collection of training and evaluation data. In contrast to the existing work, which relies on manual data collection (thus risking unintended bias) or corpora constrained in variability and limited in size (thus not allowing for reliable generalisation inferences to be made), we show how large amounts of representative data can be collected automatically using freely available tools, such as Google’s Street View, and equally automatically processed to produce a large corpus of minimally biased imagery. Using a large data set collected in the manner and comprising tens of thousands of images, we confirm our theoretical arguments that motivated our machine learning based and colour-aware histograms of oriented gradients based method, which achieved a recall of 95% and precision of 97%.

## 1. Introduction

Trees are entities of interest in many environments, which are important for a range of different applications. For example, in the context of urban planning, the distribution and placement of green spaces in general and trees in particular, is a major consideration that affects people’s satisfaction with their surroundings, mental health, overall well-being, and ability to relieve stress, as well as physical health and opportunity to engage in exercise, and so on. In so-called natural environments, such as forests and national parks, the monitoring of different tree species populations, their density, etc., are all of interest for conservation, stock evaluation, and the maintenance of ecosystem balance. In the commercial realm, trees are of major interest in agriculture, for example, for yield estimation, and in urban and suburban environments to the insurance sector, for example, in connection with storms and, in particular, tropical cyclones. The height and positioning of trees in some circumstances increase financial and other risks. In the USA, damage by falling trees alone is estimated to account for 40% of hurricane-induced cost. In other circumstances, trees have a protective role by acting as a partial block to winds, thereby reducing damage and the associated cost.

**Figure 1:**
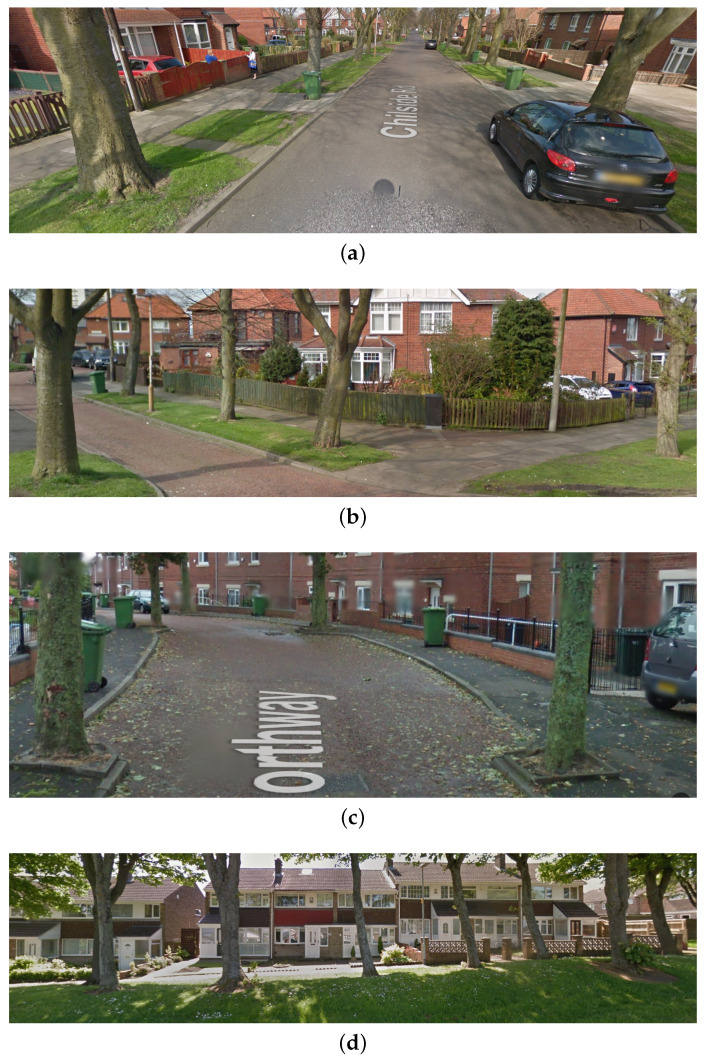
(**a**–**d**) Examples of suburban scenes of interest in the present work.

Considering the aforementioned breadth of important applications in which the location of trees plays a major role, it is hardly surprising that there has already been much research effort in this sphere. As we will detail more specifically in the next section, the first major difference between the bulk of existing work and that described in the present paper lies in the context. In short, the existing work primarily deals with either the detection of *trees* (plural) as a means of differentiating it from other flora, aerial sensing, or constrains the environment to wooded areas specifically. Herein, we are interested in the detection of individual trees in the context of suburban scenes that contain clutter highly variable in nature (buildings, cars, roads, pavements, non-tree flora, etc.), and on eye level photography only. Examples are shown in Figure 1.

The second major challenge we address, indeed for the first time in the published literature, concerns the collection of large amounts of data that is needed both for training of relevant machine learning based algorithms and, most importantly, a convincing evaluation of their performance. Specifically, a major limitation of the existing work lies in questionable test corpora, in terms of how representative they are of the breadth of real-world data on which the algorithms are supposed to be applied. So far they have been mostly manually collected, from restricted geographic areas, which raises questions of unintended bias and performance extrapolation and generalisation. Instead, we describe a principled methodology of automatically harvesting large amounts of data from arbitrary locations using a combination of Google Earth and Google Street View.

Thus, to reiterate succinctly, our main objectives concern the following two problems:

- Detection of trees from eye level photographs, and
- Automatic collection of large corpora of eye level images suitable for training and testing tree detection algorithms.

## 2. Previous Work

In this section, we review the previous work on tree detection and localisation. For completeness, we begin with approaches that use aerial views, which comprise the majority of the published literature, and then follow-up with eye level view based methods, which are closer in spirit to our work.

### 2.1. Aerial View Tree Detection

**Figure 2:**
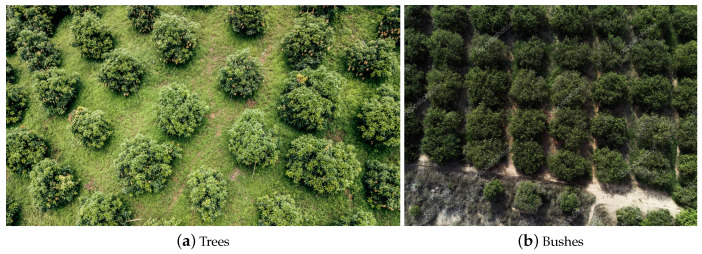
From aerial views, (**a**) tree crowns (canopies), and (**b**) bushes and other vegetation, can be very similar in appearance.

With the abundance of aerial photography freely available from sources like Google Maps and OpenAerialMap, there has been an increasing amount of research into the detection of a variety of entities of interest, such as vehicles [1], buildings [2], and roads [3] amongst others, as well as in the accurate registration of time separated imagery [4], tracking of moving objects [5,6], etc. This research has also been motivated by the increasing use of UAVs in both military and commercial applications [7]. Aerial sensing—be it in the visual spectrum, the so-called colour infrared (CIR) frequencies, or light detection and ranging (LiDAR)—has a clear appeal when it comes to tree detection given the near certainty of the absence of occlusion. Yet, the task is nevertheless a challenging one as a result of two main factors. Firstly, the appearance of trees from above can change dramatically depending on the season and it greatly depends on the specific species. Secondly, other entities such as bushes or patches of grass can be easily mistaken for trees crowns or canopies [8,9] from this angle, as illustrated in Figure 2.

Most algorithms in the literature follow a reasonably predictable and similar methodological spirit (and exhibit similar fundamental limitations), typified by a few influential works we highlight here. For example, Yang et al. [8] proposed a method that aims to perform pixel-wise tree crown detection. The classification is carried out by extracting 27 pixel level features utilising colour and local texture, combined using the Adaboost classifier.The initial pixel-wise accuracy of over 90% is increased slightly by post-processing pixel level decisions using non-local assumptions on the distribution of positive and negative decisions. Hassaan et al. [10] also adopted a multi-level approach comprising increasingly more complex discriminative steps. Firstly, *k*-means clustering of colour is used to create what is effectively a tree canopy colour model. Texture model is added next, as a way of providing further discriminative power and of distinguishing between trees and other vegetation such as grass. On a small data set (recall our prior observations regarding the representativeness of data used) they reported the accuracy of circa 70%. In addition to the aforementioned small evaluation corpus size, when the content of the images used is scrutinised, it is difficult not to doubt how generalisable these findings are when areas of different densities and distributions of trees are considered, or indeed areas with different climates and faunas.

A number of other approaches have been proposed, generally relying on simple filtering, for example, by Pouliot et al. [11], Kaartinen et al. [12], Pitkänen et al. [13], Vauhkonen et al. [14], and others.

### 2.2. Eye Level View Tree Detection

Notwithstanding a number of clear advantages of aerial sensing based approaches, tree detection from conventional, eye level view imagery remains extremely important even though the literature is numerically vastly dominated by the former group. From a practical standpoint, in some applications, only eye level images are available, for example, when detection is performed by car mounted equipment [15], say for the purpose of real-time decision-making. It is also the case that the types of flora easily confused for trees in aerial images, such as bushes as previously noted, are much easier to recognise from eye level images. Hence, this area of research has also consistently attracted research attention, more so in consumer oriented applications, rather than for those aimed at forestry, agriculture, and military.

Ali et al. [15] correctly recognised the problem posed by the variability in tree crests, which occurs both across different species as well as within the same species depending on the season and other factors. Hence, they focused on tree trunks, which exhibit lesser variability in appearance while still remaining characteristic enough to allow them to be distinguished from other semantic entities expected to be found in so-called natural images. Indeed, most of the work follows this broad idea. Thus, Ali et al. employed a combination of colour and texture features. As regards to colour, they examined a variety of common colour spaces—such as red-green-blue (RGB), hue-saturation-value (HSV), Ohta, and lightness-redness-yellowness (L*a*b*)—and different descriptors built upon these—such as co-occurrence matrices, and histograms. Similarly, with texture they considered a number of well-known texture descriptors—such as Gabor wavelets and several variations of local binary patterns. All of these are computed on small (cc. 15×15 pixels), dense, and non-overlapping image blocks, which are then classified using standard classifiers, namely a *k*-nearest neighbour classifier and off-the-shelf neural network. The authors reported promising results, though these have to be taken with a grain of salt. With reference to our observations in the previous section, the evaluation exhibits many of the usual weaknesses, namely corpus, which is rather small (cc. 100 images) and was acquired in a rather particular environment, which greatly simplifies the task (specifically, dark bark trees in snowy scenes, with significant separation of individual trees).

Yildiz [16] also focuses his attention on tree stumps and, on the conceptual level, follows a rather similar approach to that of Ali et al. [15]. Colour characteristics of tree stumps are again learnt, this time using a more expressive Gaussian mixture model. Following an expectation-maximisation applied across different patches in an image, high likelihood areas are used to extract the potential edges of trees which are post-processed to account for structural constraints of real trees. Yildiz also reports promising results that, as before, should be caveated with an observation that all of the aforementioned weaknesses of empirical evaluation of algorithms in this realm are to be observed here as well.

## 3. Proposed Methods

Unlike the existing work in the area, which universally focuses on rather constrained environments—for example, scenes in natural parks absent of any human made structures and void of clutter highly variable in type and appearance, or images in which trees are highly separated from potentially confounding semantic elements—our work targets much more challenging suburban environments that are rife with clutter and unpredictability. These scenes may contain houses, fences, streets and street signs, cars and other motorised vehicles, bicycles and pedestrians, and a host of other unknown elements. In order to approach such a demanding problem in a systematic fashion, we describe two algorithms. The first of these follows the present zeitgeist in the field and uses what may be called traditional computer vision approaches, which make extensive use of domain knowledge in the model building process. The second algorithm is rather different in nature. While it too has a bottom-up structure, it places a much heavier burden on automatic machine learning. The idea is that of employing general (rather than highly domain specific) low level features and then using annotated training data to learn how to utilise these to make inferences about more complex visual structures.

### 3.1. First Approach: Explicit Shape Constrained Statistical Appearance Model

**Figure 3:**
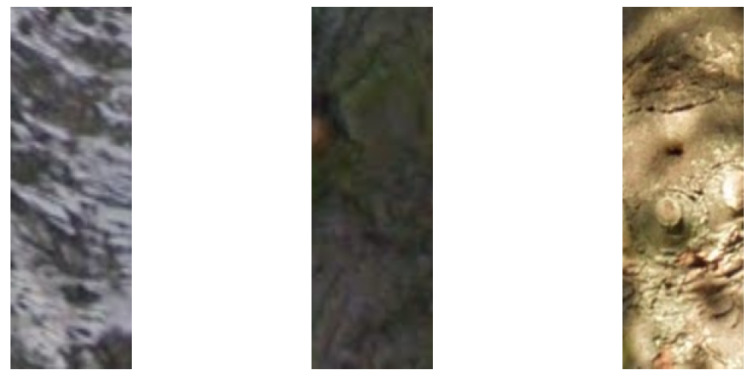
The appearance of tree bark, both in terms of its texture and colour, is highly discriminative but also highly variable across species.

As noted in Section 2.2, the appearance of tree bark is highly characteristic, as illustrated in Figure 3. We too make use of this observation and use colour as the first low level feature as a means of quickly rejecting image areas that are not of interest. We adopt the use of the Gaussian mixture model as a flexible yet tractable way of modelling tree bark colour. We train the model using what is initially a set of a couple of hundred hand segmented images of tree bark and which are synthetically augmented with simple random photometric transformations [17] to produce more than a thousand training images.

The model is applied densely on a pixel by pixel basis. We used the Mahalanobis distance, which emerges naturally from the nature of the statistical model employed and is hence most frequently used in the literature [18,19], which for computational reasons we also use as the threshold, thus removing from further consideration those image loci that are clearly purely described by the learnt model. Lastly, in order to eliminate unavoidable noise in the output, given that our processing was highly local in nature (pixel wise), we perform morphological based post-processing comprising opening and closing operations on the initial output.

#### Contour Consistency

As suggested by previous work, the colour of tree bark alone appears to be quite successful in its own right. Nevertheless, it is clear that greater robustness to different scenes, tree species, etc., can be achieved by using simple structural constraints that characterise trees. Thus, our idea is to eliminate further spurious detections of potential stumps by requiring continuous bark textured regions also to form tree like structures. We achieve this by combining our colour based model with image edge based information.

**Figure 4:**
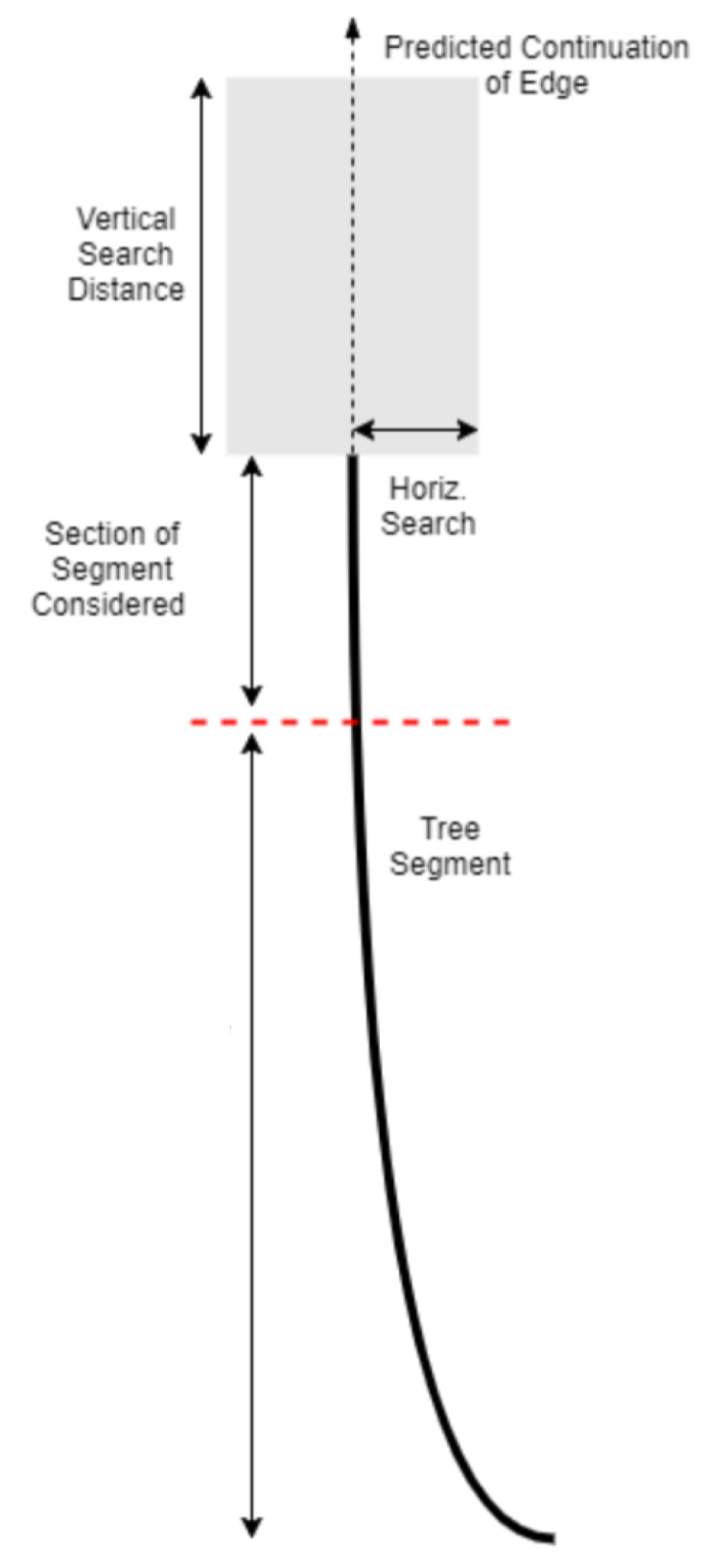
Concatenation of stump segments.

**Figure 5:**
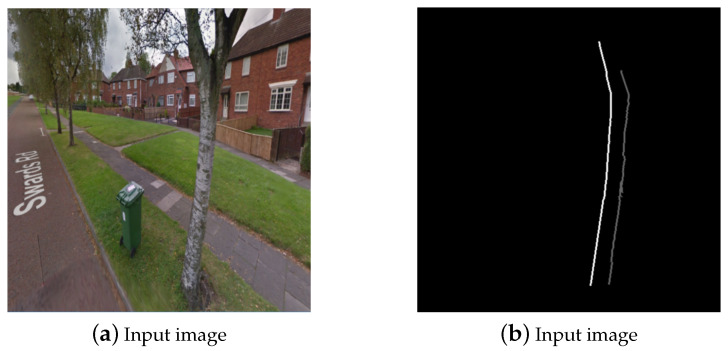
(**a**,**b**) Example of tree stump detection and delineation using our first conventional computer vision based method.

In particular, we start by detecting Gabor edges [20]. Then we are able to identify those edges that correspond to tree edges by finding pairs of edges that enclose high tree likelihood regions under our colour model. This process bootstraps a more complex post-processing algorithm that now uses what are effectively tree stump segments to connect them, thus forming a longer and more reliable continuous tree stump, which can then exhibit greater flexibility in shape. Specifically, by taking into account both colour based likelihood and the geometric directionality of segments, as well as stump width consistency, disjoint segments can be inferred to belong to the same tree and thus connected with each other—a process that is repeated until no further joining is possible; see Figure 4 for a conceptual illustration and Figure 5 for a visual example.

### 3.2. Second Approach: SVM Based Learning over Bag of Visual Words

Our first method followed the spirit of the bulk of existing research, which relies on heavy engineering and extensive use of domain knowledge emerging from human understanding of the semantics of the content of interest. Conceptually this can be thought to be akin to rule based expert systems. While such approaches have their place and application in the right circumstances, they generally lack the required level of robustness and flexibility in the realm of problems with complex underlying structures in which explicit rules and building blocks are difficult to identify or specify. Indeed, as our experiments (elaborated on in the next section) demonstrate, this is unsurprisingly (recall all the complexities highlighted in Section 2.2) the case in the problem under consideration here too. Thus, the second method we introduce here takes a different approach, making less use of prior, human knowledge and domain specificity, and instead allowing for greater flexibility by placing a much heavier burden on learning from appropriate but semantically minimally meaningful elementary features.

The “bag of words” model has now been put to use in an extensive number of diverse applications. Originally formulated for use in textual document related problems (classification, retrieval, relatedness, etc.) [21,22] it has since been adapted for use in audio [23] (“bag of audio words”), shape recognition [24] (“bag of boundaries”), image based [24], and video based object recognition [25] (“bag of visual words”). Unlike in text processing where elementary semantic primitives (i.e., words) impose themselves naturally, in audio or image and video analysis, primitives are learnt in an unsupervised fashion. The general approach comprises the following steps:

(1) Elementary local (be it in space, time, or space-time) features are extracted from a training data corpus.
(2) Clustering in the feature space is used to learn a segmentation of the space into regions, each of which is deemed to correspond to an abstract “word” (hence, e.g., audio or visual words).

In computer vision, a popular example of elementary features are scale invariant features (SIFT), extracted over a dense grid or at automatically detected salient interest points. Another popular choice comes in the form of histograms of oriented gradients (HOGs), which too can be extracted densely or sparsely. Clustering is usually performed using simple *k*-means. The optimum number of clusters, *k*, is dependent not only on the domain of application but also the nature of the problem, for example, retrieval or classification. In the case of the former, usually a large vocabulary is used [26] whereas in the latter, a smaller one, providing greater generalisation [27]. The problem considered in the present paper clearly falls in the latter group; hence, we select the value of k=200, which is in line with previous work on other problems of the structurally-similar kind.

Then, just as documents may holistically be represented by histograms over a vocabulary of actual words, audio recordings, images, or videos may be represented as histograms over the corresponding audio or visual words. Indeed, we adopt the same approach but considering that the objects (tree stumps) of interest, if present, are in unknown locations in input images, and have a limited spatial extent, it is image patches that we represent using histograms over visual word vocabularies. Given its successes over a range of different problems, both when applied sparsely and densely, we adopt the use of SIFT as the elementary local feature.

**Figure 6:**
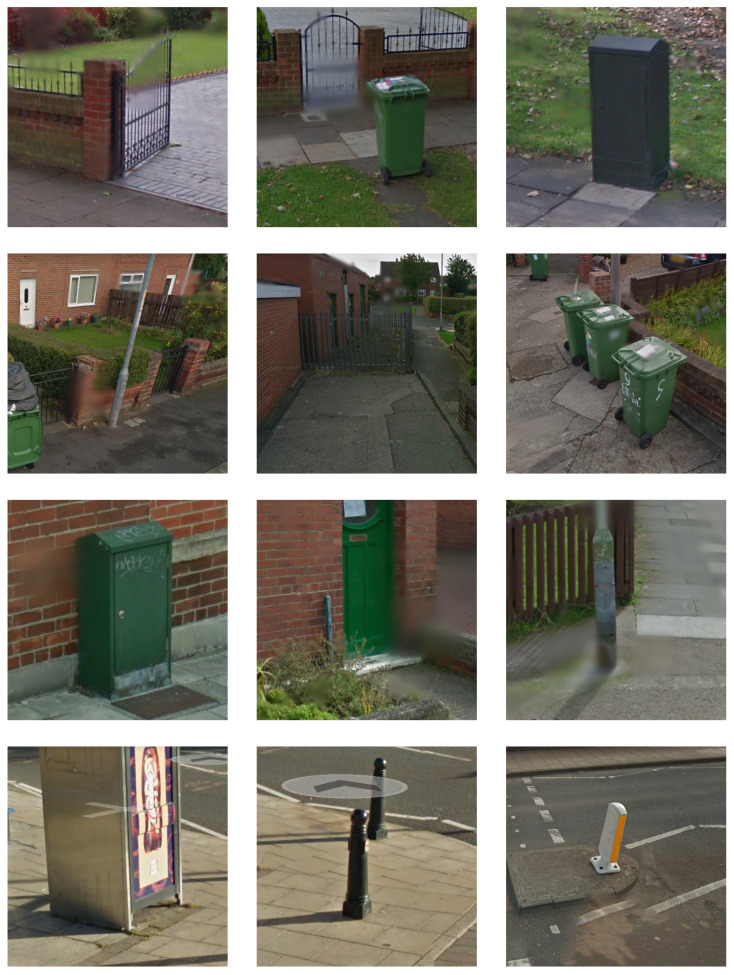
Examples of training images not containing tree stumps.

**Figure 7:**
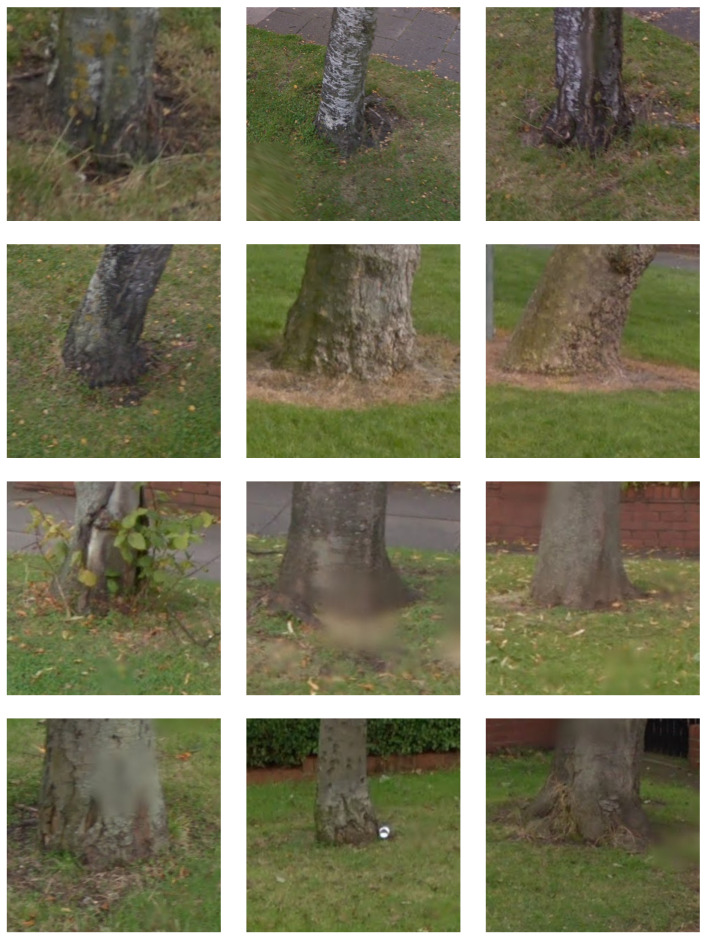
Examples of training images containing tree stumps.

Finally, to facilitate learning over image patch descriptors in the form of visual word histograms, we employ a linear support vector machine; thus, henceforth we will refer to this method as BoVW-SVM(lin). Our choice is again motivated by findings stemming from previous research and the impressive performance of this specific classifier on arguably similar problems with the same kind of local features underlying the learning process [28]. Figure 6 and Figure 7 show a small but representative sample of images used for training.

### 3.3. Third Approach: SVM Based Learning over Colour Aware HOGs

Following the introduction of our first method, which bears the greatest similarity to the existing methods in the literature, we argued against highly engineered, human domain knowledge reliant approaches. Thus, we proposed an alternative, which places greater emphasis on data drive learning from elementary visual features, in our case SIFT features extracted over a dense grid. Notwithstanding the aforementioned advantage of the latter method, we hypothesised (and subsequently empirically verified) that the lack of use of colour information causes a significant loss of important information—colour is an important characteristic that aids in distinguishing *tree* stumps from various man-made objects, such as lamp posts and the like. Our third approach seeks to remedy this and make the most use both of data drive supervised learning and unsupervised learning of colour.

As in the previous method, we use a pyramidal structure of sliding windows, each of which we seek to classify as either containing or not containing a tree stump. However, unlike before, we extract local features not from greyscale patches but rather from each of the three colour channels. Furthermore, to make the best use of colour information, we do not work in the default red-green-blue (RGB) colour space but rather luma-chrominance (YUV). As demonstrated in a wide range of applications, colour space transformations can affect performance greatly [29]. Our choice of YUV is motivated both by previous findings in the literature as well as its human perception orientation [30,31]. Recall that YUV representation can be obtained from RGB by means of a linear transformation. Different variations thereof exist [32], and herein, we adopt one of the more common ones:

(1) $$\begin{array}{c} \left[\begin{array}{c} Y\\ U\\ V \end{array}\right]=\left[\begin{array}{ccc} 0.299 & 0.587 & 0.114\\ -0.14713 & -0.28886 & 0.436\\ 0.615 & -0.51499 & -0.10001 \end{array}\right]\left[\begin{array}{c} R\\ G\\ B \end{array}\right] \end{array}$$

Moreover, we considered the use of two different types of local features applied on the colour channels: namely SIFT and HOGs. An a priori argument can be made for the superiority of either of these. Namely, it is plausible that the rotational quasi-invariance of SIFT is a desirable property. However, it is equally sensible to make the exact opposite argument, given that bark texture in many cases exhibits stark anisotropicity. Our experiments demonstrated the choice of the elementary feature indeed does matter and does so greatly, with HOGs significantly outperforming SIFT. Hence, the former was adopted in the present method for all subsequent experiments. As before, we adopt an SVM, trained in a supervised fashion, to classify image patches as containing or not containing a tree stump. However, unlike before, we use a radial basis function based kernel in order to better capture the additional complexity introduced by the more complex representation proposed; accordingly, we will henceforth refer to this method as HOG-SVW (RBF).

## 4. Empirical Evaluation

In this section, we summarise our experimental methodology, results, and findings. We begin with another technical contribution, which addresses the problem of automatically collecting large amounts of training and test data, crucial for the convincing and insightful evaluation of the problem under consideration.

### 4.1. Automatic Harvesting of Big Data

**Figure 8:**
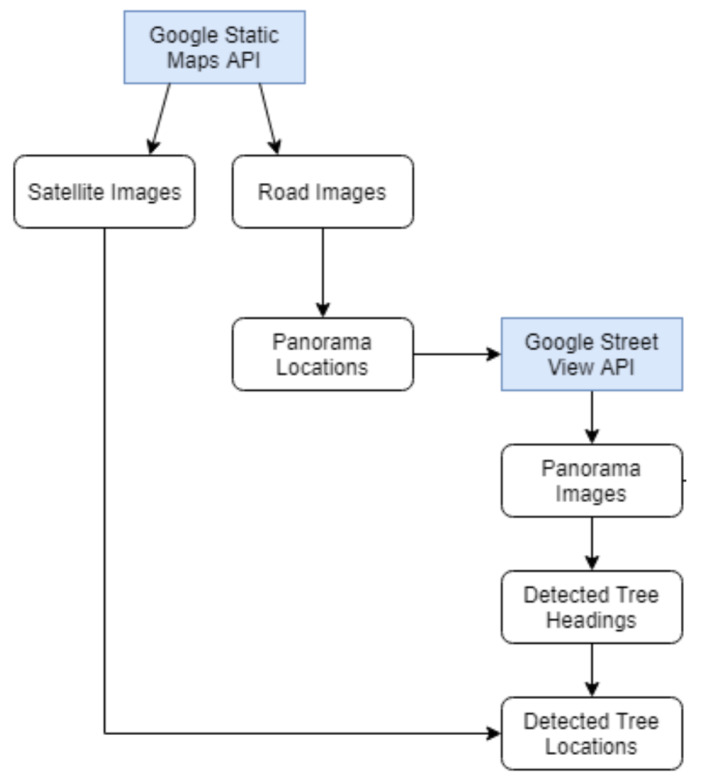
Overview of our method for automatic data harvesting.

In Section 2.2, we highlighted as a major methodological problem of research in the existing literature the quality of data used for evaluation. As noted then, these include corpora, which are highly limited in size or constrained to specific environments, all of which limit conclusions that can be drawn about the ability of different methods to generalise across different contexts, seasons, habitats, species, etc. Herein we address this problem by describing how massive amounts of data can be harvested automatically using a range of freely available Google tools. An overview is conceptually depicted by the diagram in Figure 8.

**Figure 9:**
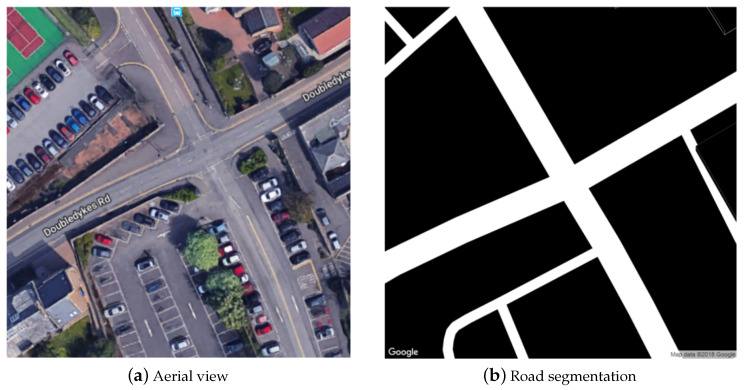
An example of (**a**) Google Maps aerial view of a suburban land area, and (**b**) the corresponding road segmentation.

Recall that unlike most research in the published literature, our work focuses on the highly challenging task of tree detection in suburban environments in which clutter is ubiquitous and highly variable in appearance, scale, and type. Our idea is to use Google’s Street View to obtain relevant imagery. In particular, we employ Static Maps API to request a segmentation of a specified land area into road and non-road regions, as shown in Figure 9. Using this data allows us to fetch Street View images by virtually traversing and sampling different roads.

**Figure 10:**
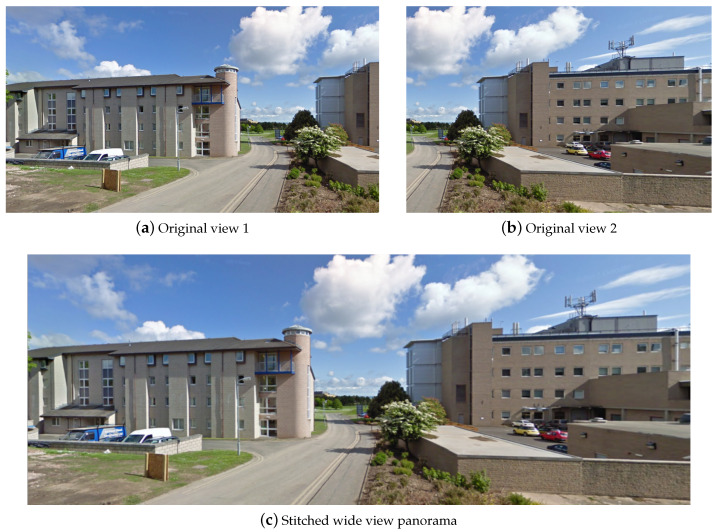
An example of (**a**,**b**) a pair original Street View images, and (**c**) the panoramic image resulting from stitching them together.

The next practical challenge we encountered concerns the size of fetched images, each of which is limited to the maximum of 640×640 pixels. This generally corresponds to quite narrow views and, thus, often trees that are not fully contained within any one image available ‘off-the-shelf’. To overcome this problem, from the same location we harvested multiple images of the scene viewed from different directions, and stitched these into a single, wide view panoramic image. This is illustrated in Figure 10. Subsequent tree detections also allow to compute the exact tree locations via simple triangulation from multiple views and thus, if desired, also collect the corresponding aerial view imagery too; see Figure 8. For our evaluation, we used circa 10,000 panoramic images, formed from a much greater number of original narrow view Street View scenes.

### 4.2. Results and Discussion

We started our evaluation in the order in which we introduced our method, that is, with the approach introduced in Section 3.1 which bears the greatest similarity to the majority of methods in the existing literature. Recall that we made arguments from theory why this approach is not best suited to the problem considered in this work. However, even we were struck with just how poorly the method performed in practice; that is, on our data as compared with scenes in less challenging conditions. Indeed, the performance was found to be so poor that we saw little point in performing much analysis. To ensure that there was no mistake on our behalf (though we had already seen the algorithm work reasonably on simple scenes), we also ran the original method of Yildiz [16] and observed similar behaviour. This finding provides strong evidence for our hypothesis that highly domain specific approaches, which rely on heavily manually-engineered steps with reliance on explicitly encoded human knowledge, are at best suited only to constrained environments examined in the previous work (wooded areas, uncluttered scenes with clear tree separation, etc.) but not highly variable ones such as those considered in the present paper.

We thus turned our attention to the second two methods introduced in Section 3.2 and Section 3.3, which use minimal manual engineering (constrained to the elementary features only, and even this being highly biologically inspired in nature) and instead place much greater importance on automatic learning. As expected, both performed far better than the first method (it would have been difficult to do worse), though there was a significant difference between the two, as we shall shortly see.

A summary of the results is shown in Table 1. It can be seen that the bag of visual words based method achieved a very good recall rate of 0.85 albeit with rather poorer precision of 0.39 due to the high false acceptance rate (i.e., tree stumps being identified where there were none). By far the best performance was demonstrated by our colour aware histograms of oriented gradients based method, which achieved a recall of 0.95 and outstanding prevision of 0.97, with a false acceptance rate of only 0.03. These findings confirm our hypothesis regarding the use of colour, which motivated the approach.

Further insight can be gained by examining the corresponding confusion matrices in Table 2a,b. Though at first sight the two look similar, with the colour aware histograms of oriented gradients based method achieving notably higher true positive rate for the tree stump class, it is important to take note of the high class imbalance: in suburban scenes there were far more regions that do not contain trees. Thus, the seemingly small difference in the false positive negative rate for the non-tree class is actually significant in the fullness of the recognition context.

## 5. Summary and Conclusions

The goal of this work was to detect trees in eye level view images in challenging suburban scenes rich in clutter, which is highly variable in type and appearance, comprising such entities such as houses, falls, shrubs, cars, bicycles, pedestrians, hydrants, and lamp posts. This setting is in sharp contrast to the existing work on tree detection, which either uses aerial imagery, highly constrained environments, such as natural parks and wooded areas, or simple scenes with little clutter and clear tree separation.

We motivate and introduce three different approaches. The first of these bears the greatest semblance to the methods that dominate the existing literature and can be seen as employing conventional computer vision that makes extensive use of explicit human knowledge. Unlike in the simpler context described above, on our highly challenging data set, the method was found to perform extremely poorly. We explained that this was expected from theory, which is why we argued for more flexible, machine learning oriented approaches. Thus, we next introduced an algorithm that uses densely extracted local SIFT features, collected in a bag of visual words, and mapped into tree stump and not tree stump classes, using a linear support vector machine. In line with our theoretical expectations, this method performed far better in challenging conditions, achieving high recall rates, albeit with an excessively high false acceptance rate. This too was anticipated in our prior theoretical discussion, which is why we proposed our third and most novel method, which makes use both of colour and characteristic tree bark texture. It does so by employing histograms of oriented gradients on YUV colour channels, with the ultimate decision of whether an image patch corresponds to a tree stump, being made with a radial basis functions kernel based support vector machine. This approach demonstrated the most impressive performance, achieving recall in excess of 95% and precision in excess of 97%, far outperforming the existing methods in the literature. What is more, we trust that our analysis and discussion will further benefit the relevant research community by highlighting some of the methodological challenges of the previous work as well as the proposed means of addressing them, as well as by directing further improvements and research effort.

## Figures and Tables

**Table 1:** Comparison of our Approach 1, which utilises a dense grid based bag of scale invariant features (SIFT) visual words, and Approach 2, which instead uses a dense based colour aware histogram of oriented gradients.

Method	Precision	False Acceptance	Recall
BoVW-SVM (lin)	0.39	0.61	0.85
HOG-SVM (RBF)	0.97	0.03	0.95

**Table 2:** Confusion matrices for (**a**) the bag of visual words based method of Section 3.2, and (**b**) the colour aware histograms of oriented gradients (HOGs) based method of Section 3.3. N.b. The false negative rate for the non-tree class is not exactly 0 but 0.00 to the precision of two significant digits.

**(a) BoVW-SVM (lin)**		
**True**\**Predicted Label**	**Tree**	**Non-Tree**
Tree	0.85	0.15
Non-tree	0.03	0.97
**(b) HOG-SVM (RBF)**		
**True**\**Predicted Label**	**Tree**	**Non-Tree**
Tree	0.95	0.05
Non-tree	0.00	1.00

## References

[1] Tang T., Zhou S., Deng Z., Lei L., Zou H. (2017). Arbitrary-oriented vehicle detection in aerial imagery with single convolutional neural networks. Remote Sens..

[2] Paparoditis N., Cord M., Jordan M., Cocquerez J.P. (1998). Building detection and reconstruction from mid-and high-resolution aerial imagery. Comput. Vis. Image Underst..

[3] Sirmacek B., Unsalan C. (2010). A probabilistic framework to detect buildings in aerial and satellite images. IEEE Trans. Geosci. Remote Sens..

[4] Arandjelović O., Pham D.S., Venkatesh S. (2015). Efficient and accurate set-based registration of time-separated aerial images. Pattern Recognit..

[5] Li S., Yeung D.Y. Visual object tracking for unmanned aerial vehicles: A benchmark and new motion models. Proceedings of the AAAI Conference on Artificial Intelligence.

[6] Arandjelović O. Automatic vehicle tracking and recognition from aerial image sequences. Proceedings of the IEEE International Conference on Advanced Video and Signal Based Surveillance.

[7] Lee S. (2017). Aerial Vehicle. U.S. Patent.

[8] Yang L., Wu X., Praun E., Ma X. Tree detection from aerial imagery. Proceedings of the ACM SIGSPATIAL International Conference on Advances in Geographic Information Systems.

[9] Gomes M.F., Maillard P. (2016). Detection of Tree Crowns in Very High Spatial Resolution Images.

[10] Hassaan O., Nasir A.K., Roth H., Khan M.F. (2016). Precision forestry: Trees counting in urban areas using visible imagery based on an unmanned aerial vehicle. IFAC-PapersOnLine.

[11] Pouliot D., King D., Bell F., Pitt D. (2002). Automated tree crown detection and delineation in high-resolution digital camera imagery of coniferous forest regeneration. Remote Sens. Environ..

[12] Kaartinen H., Hyyppä J., Yu X., Vastaranta M., Hyyppä H., Kukko A., Holopainen M., Heipke C., Hirschmugl M., Morsdorf F. (2012). An international comparison of individual tree detection and extraction using airborne laser scanning. Remote Sens..

[13] Pitkänen J., Maltamo M., Hyyppä J., Yu X. (2004). Adaptive methods for individual tree detection on airborne laser based canopy height model. Int. Arch. Photogramm. Remote Sens. Spat. Inf. Sci..

[14] Vauhkonen J., Ene L., Gupta S., Heinzel J., Holmgren J., Pitkänen J., Solberg S., Wang Y., Weinacker H., Hauglin K.M. (2012). Comparative testing of single-tree detection algorithms under different types of forest. Forestry.

[15] Ali W., Georgsson F., Hellstrom T. Visual tree detection for autonomous navigation in forest environment. Proceedings of the IEEE Intelligent Vehicles Symposium.

[16] Yıldız T. (2010). Detection of Tree Trunks as Visual Landmarks in Outdoor Environments. Ph.D. Thesis.

[17] Zachariou M., Dimitriou N., Arandjelović O. (2020). Visual Reconstruction of Ancient Coins Using Cycle-Consistent Generative Adversarial Networks. Science.

[18] Arandjelović O. Making the most of the self-quotient image in face recognition. Proceedings of the IEEE International Conference and Workshops on Automatic Face and Gesture Recognition.

[19] Arandjelovic O., Cipolla R. An illumination invariant face recognition system for access control using video. Proceedings of the British Machine Vision Conference.

[20] Mehrotra R., Namuduri K.R., Ranganathan N. (1992). Gabor filter-based edge detection. Pattern Recognit..

[21] Choudhary B., Bhattacharyya P. Text clustering using semantics. Proceedings of the International World Wide Web Conference.

[22] Beykikhoshk A., Arandjelović O., Phung D., Venkatesh S. Overcoming data scarcity of Twitter: Using tweets as bootstrap with application to autism-related topic content analysis. Proceedings of the International Conference on Advances in Social Networks Analysis and Mining.

[23] Pancoast S., Akbacak M. Bag-of-audio-words approach for multimedia event classification. Proceedings of the Annual Conference of the International Speech Communication Association.

[24] Arandjelovic O. (2013). Matching objects across the textured-smooth continuum. arXiv.

[25] Rieutort-Louis W., Arandjelović O. Descriptor transition tables for object retrieval using unconstrained cluttered video acquired using a consumer level handheld mobile device. Proceedings of the International Joint Conference on Neural Networks.

[26] Sivic J., Zisserman A. Video Google: A text retrieval approach to object matching in videos. Proceedings of the IEEE International Conference on Computer Vision.

[27] Fare C., Arandjelović O. Ancient roman coin retrieval: A systematic examination of the effects of coin grade. Proceedings of the European Conference on Information Retrieval.

[28] Bristow H., Lucey S. (2014). Why do linear SVMs trained on HOG features perform so well?. arXiv.

[29] Arandjelović O. (2012). Colour invariants under a non-linear photometric camera model and their application to face recognition from video. Pattern Recognit..

[30] Tsai T., Huang Y.P., Chiang T.W. Image retrieval based on dominant texture features. Proceedings of the 2006 IEEE International Symposium on Industrial Electronics.

[31] Sudhir R., Baboo L.D.S.S. (2011). An efficient CBIR technique with YUV color space and texture features. Comput. Eng. Intell. Syst..

[32] Palus H. (1998). Representations of colour images in different colour spaces. The Colour Image Processing Handbook.

